# Application of Gas Sensor Arrays in Assessment of Wastewater Purification Effects

**DOI:** 10.3390/s150100001

**Published:** 2014-12-23

**Authors:** Łukasz Guz, Grzegorz Łagód, Katarzyna Jaromin-Gleń, Zbigniew Suchorab, Henryk Sobczuk, Andrzej Bieganowski

**Affiliations:** 1 Faculty of Environmental Engineering, Lublin University of Technology, Nadbystrzycka 40B Str., Lublin 20-618, Poland; E-Mails: l.guz@wis.pol.lublin.pl (Ł.G.); z.suchorab@wis.pol.lublin.pl (Z.S.); h.sobczuk@wis.pol.lublin.pl (H.S.); 2 Institute of Agrophysics, Polish Academy of Sciences, Doswiadczalna 4 Str., Lublin 20-290, Poland; E-Mails: k.jaromin-glen@ipan.lublin.pl (K.J.-G.); a.bieganowski@ipan.lublin.pl (A.B.)

**Keywords:** gas sensor array, electronic nose (e-nose), sewage physical-chemical parameters, wastewater treatment, sequencing batch reactors (SBR)

## Abstract

A gas sensor array consisting of eight metal oxide semiconductor (MOS) type gas sensors was evaluated for its ability for assessment of the selected wastewater parameters. Municipal wastewater was collected in a wastewater treatment plant (WWTP) in a primary sedimentation tank and was treated in a laboratory-scale sequential batch reactor (SBR). A comparison of the gas sensor array (electronic nose) response to the standard physical-chemical parameters of treated wastewater was performed. To analyze the measurement results, artificial neural networks were used. E-nose—gas sensors array and artificial neural networks proved to be a suitable method for the monitoring of treated wastewater quality. Neural networks used for data validation showed high correlation between the electronic nose readouts and: (I) chemical oxygen demand (COD) (*r* = 0.988); (II) total suspended solids (TSS) (*r* = 0.938); (III) turbidity (*r* = 0.940); (IV) pH (*r* = 0.554); (V) nitrogen compounds: N-NO_3_ (*r* = 0.958), N-NO_2_ (*r* = 0.869) and N-NH_3_ (*r* = 0.978); (VI) and volatile organic compounds (VOC) (*r* = 0.987). Good correlation of the abovementioned parameters are observed under stable treatment conditions in a laboratory batch reactor.

## 1. Introduction

The physical and chemical parameters of effluents from wastewater treatment plants (WWTP) or reclaimed water should comply with the standards described in the appropriate regulations [1]. The quality of wastewater can be determined by the measurement of many parameters, but the basics are biological oxygen demand (BOD), chemical oxygen demand (COD), pH, total organic carbon (TOC), total suspended solids (TSS), oxygen uptake rate (OUR), and levels of phosphorus and nitrogen compounds [2]. Until now, complete, continuous, and automatic WWTP monitoring systems have not been sufficiently developed due to the harsh environment and lack of reproducibility and reliability of the available sensors in such conditions. Therefore the continuous improvement of existing methods and development of new methods and systems can be seen [2].

One of these new measuring systems is the so-called electronic nose (e-nose). The e-nose imitates the human olfactory system, and consists of several crucial elements: non-selective gas sensor array, signal processing systems, and multidimensional pattern recognition engine [3]. Some details regarding the e-nose can be found in Gardner's book [4].

The e-nose has been used in many fields, but mainly in stable indoor applications. The e-nose was also successfully implemented in the medical industry [5–9]. Very promising for this purpose seem to be biosensors [10]. In addition, the e-nose can be practically applied in the pharmaceutical industry [11], cosmetology [12], the food industry [13,14] and other areas [15]. A comprehensive list of e-nose applications can be found in Wilson's article [16].

From the reviews of articles concerning the e-nose, it can be noticed that its environmental applications are limited, mainly due to unstable and variable conditions. Despite these impediments, e-nose systems are used to evaluate miscellaneous parameters of environmental sources of malodours and air pollution such as landfill sites, waste disposal, incineration plants, composting plants, and livestock farms, as described in Table 1.

Some authors have attempted to compare the standard parameters of sewage to an e-nose response. Review of these results has been shown in Table 2. Most frequently it was the evaluation of the e-nose system to recognition and classification of sewage odours concerning their location in WWTP as well as the odour concentration assessment. In some research, attempts were made to employ the e-nose for the assessment of standard physical-chemical parameters of sewage such as COD, TSS, volatile suspended solids (VSS) and turbidity, but the correlation coefficient was weak (*r* = 0.52 ÷ 0.67) [17]. It was also possible to determine the BOD of sewage.

Because these studies indicated that the e-nose systems could be used to monitor sewage parameters, a second step was made. The e-nose systems started to serve as an early warning system against intermittent or accidental discharge of abnormal substances into the sewer systems or WWTP. Mentioned substances, e.g., oil derivatives or other chemical substances, could be barely biodegradable, or could be poisonous to the active sludge/biofilm and cause a malfunction of, or a cost increase in, the bioreactor's performance. Considering the large amount of polluting compounds, a system applied with many low selective gas sensors seems to be suitable for detecting any possible incidents. Such a prototype system was installed in the WWTP at Cranfield University. The e-nose has detected episodes of sewage tainted with diesel or unknown pollutants [35,36]. During monitoring in the WWTP mentioned, dilution of sewage caused by heavy rain was recorded. The system can also detect deliberately introduced 5 ppm 2-chlorophenol, methylisoborneol and 2-chloro-6-methylphenol [37]. It was also able to inform about the status of the device, such as main pump failure due to sewage parameter changes [45].

The abovementioned review of scientific articles confirms the dependence between gas sensor array readouts and the chemical-physical parameters of wastewater (liquid phase). Additionally, nowadays there are commercially available devices that determine BOD on the basis of the analysis of air over a wastewater level. As an example of such devices it could be mentioned the OxiTop^®^ WTW, which is a respirometer that determines BOD according to DIN EN 1899-2 (H55) water quality—determination of biochemical oxygen demand after *n* days (BODn). Owing to the fact that, in wastewater with a consistent composition, levels of BOD and COD parameters are usually proportional, it can be concluded that it is possible to extend this technique for COD determination and other Parameters classically used in establishing wastewater parameters. However, for analysis of air headspace over wastewater levels it an e-nose was used as a tool applicable not only for carbon compounds, but also other pollutants.

It seems obvious that, when referring to a liquid medium, an electronic tongue would be employed instead of an electronic nose. Additionally, parameters such COD, TSS, turbidity, and nitrogen compounds can be successfully determined using traditional, submerged sensors, with well-established technical status. Regardless, the major goal of this work was to attempt to find whether the analysis of the gaseous phase gives the opportunity to precisely estimate the composition of the liquid phase, in the context of the determination of particular parameters of treated wastewater. To conduct the presented research the authors were also encouraged by the results achieved by other scientists, as presented in Table 2, which were connected not only with odor concentration in WWTP devices but also the parameters of wastewater expressed as standard contamination indicators, e.g., BOD, COD, TSS.

The above-described investigations show that e-nose systems can be successfully used for early warning when hazardous substances (for the purification process) appear in the sewage. Therefore, at the next stage, use of the e-nose system for monitoring the process of wastewater purification in the WWTP can be expected. However the review of the literature shows that there has been a lack of such investigations until now.

The aim of this paper is to describe the e-nose system (consisting of a matrix of non-selective gas sensors and artificial neural networks) and its use, to monitor the conformity of the effect of wastewater treatment by activated sludge in a sequencing batch reactor (SBR). To achieve the aim authors compared the e-nose results of sewage headspace measurements in the SBR to the selected standard physical-chemical parameters of wastewater.

## 2. Experimental Section

For the measurement of wastewater quality, traditional physical-chemical methods were used, together with an alternative that consisted of headspace analysis using e-nose. The results of these methods were compared afterwards. During the experiments two main datasets were recorded, which enabled evaluation of the non-specific gas sensor array for assessment of the quality of wastewater treated in an SBR reactor through the activated sludge method. The first dataset consisted of the gas sensors array measurement results of quiescent sewage liquor headspace. The second dataset refers to the quality of a treated wastewater sample, collected from the bioreactor chamber at the end of sedimentation phase, which occurs immediately after headspace sampling. Because the time interval between the end of the sedimentation phase and this series of samples was very short, the obtained results should be treated as the description of the last moments of sedimentation and not a description of the decantation phase.

Measurements were conducted during the correct performance of the SBR bioreactor. Authors did not test the system when sewage quality varied significantly in time. This work presents the measurement results for a stable treatment process in a laboratory-scale bioreactor of wastewater, where wastewater was taken from the same site, after mechanical treatment in this same municipal WWTP. The sewage did not contain poisonous substances that could significantly disturb the treatment process. In order that a significant quality change of the sewage considered would only occur in case of dosing of poisonous substances to the laboratory bioreactor, or intentionally disturbing the performance of important systems like aeration or mixing. But these circumstances were not considered by the authors.

E-nose measurements of wastewater parameters are possible by Henry's law. At a constant temperature, the concentration of specific compounds in the gaseous phase *C_G_*, being in equilibrium with liquid, is directly proportional to the concentration in the liquid phase. This relationship is described by equation H = C_G_/C_V_ and allows assessment of a concentration of organic and mineral compounds in wastewater by performing a headspace analysis. These compounds directly or indirectly affect wastewater parameters such as COD and concentration of nitrogen compounds, as well as turbidity and TSS.

### 2.1. Sequencing Batch Reactor

The SBR is a suitable facility to be measured using a gas sensor array—this also concerns chemical as well as biological reactors that are used for wastewater treatment. All treatment processes are performed in one volume in the proper sequence and, because of this, only one measuring device is sufficient for monitoring. In the classical WWTP working in flow mode, to collect comparable data about the following process of purification, the measurements should be conducted in several places.

The laboratory-scale SBR reactor used for the experiment treated sewage with activated sludge in a 12 h operating cycle. Sewage used in the experiment was collected in a primary sedimentation tank of a municipal WWTP located in Lublin-Hajdów (South-eastern Poland). From the second hour of the SBR cycle, lasting 9 h, continuous mixing started. Simultaneously, together with mixing, continuous aeration was activated for a period of 2.5 h, and then intermittently in order to sustain oxygen concentration at the level of 2 gO_2_/m^3^. In the final part of the cycle, a 2 h sedimentation phase was initiated and finished with decantation, during which the treated wastewater was discharged and after which the reactor was loaded with raw sewage. The SBR reactor enabled reduction of organic compounds and biogene concentration (compounds of carbon, nitrogen and phosphor) during an active phase of treatment mixing and aeration. The laboratory equipment consisted of three independent SBR reactors, each with a 10 dm^3^ chamber. All reactors were equipped with a mechanical stirrer, aeration system with membrane diffuser, and temperature stabilization system. During the experiment, the temperature of sewage was kept at 20 ± 0.1 °C.

### 2.2. E-Nose

During selection of the gas sensors, the following criteria were taken into consideration: (I) low susceptibility to humidity and temperature; (II) possibly low number of sensors—less complexity and lower cost of measurement device; (III) non-specific or low-selective sensors; (IV) gas sensors tested in a similar measurement system and commonly available in most countries; (V) relatively low power consumption (the ability to use in a portable device); and (VI) the same type of sensors in an array.

The e-nose used in the research contains eight metal oxide semiconductor (MOS)-type gas sensors. TGS Figaro sensors were implemented, as detailed in Table 3. These are relatively small sensors (TO-5 metal can package) with a power consumption up to 300 mW. Each sensor gives a different signal response according to its sensitivity characteristics. In addition, Dallas DS18B20 and Honeywell HIH-4000 sensors were used to measure gas temperature and relative humidity, respectively. These enabled compensation of temperature and humidity influence on the gas sensors' response. Signals from all sensors (8 + 2) created an array response.

Resistive MOS are used for gas detection, due to changes in the electrical properties of sensing elements as a result of surface chemical reactions between gas molecules and the semiconductor, proportional to gas concentration. The measuring element of the gas sensor is made of the sintered tin dioxide SnO_2_, which reveals a significant increase in conductivity in terms of reducing gas atmosphere. According to the basic measuring circuit, recommended by the manufacturers, to measure the sensor resistance a simple resistive voltage divider can be used. The resistive sensor (connected to a reference voltage *V_C_*) is placed in series with a load resistor *R_L_* (connected to the ground of the circuit) [46]. The resistance of the sensor is determined according to the equation: *R_S_* = *R_L_* · (*V_C_* − *V_OUT_*) · (*V_OUT_*)^−1^, where *R_S_* resistance of the sensor [Ω], *R_L_* resistance of load resistor [Ω], *V_C_* voltage reference of resistor divider [V], *V_OUT_* output voltage of resistor divider [V]. The array response signal was measured using the 24-bit analogue to digital converter of the ADuC847 microcontroller.

**Figure 1.**
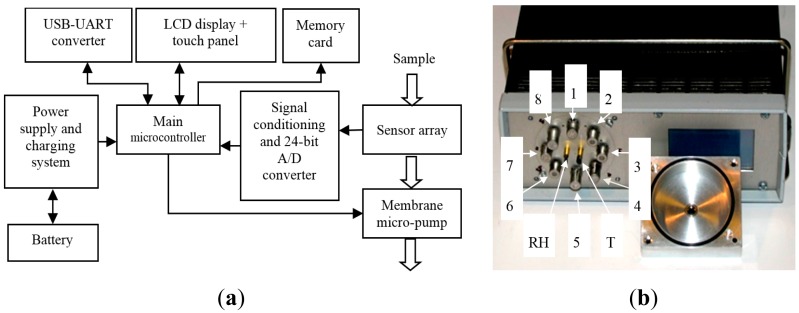
(**a**) Construction of the e-nose and laboratory setup: block diagram of device; (**b**) Construction of the e-nose and laboratory setup: sensors array visible after dismantling of sensor chamber front cover: 1—TGS2600-B00, 2—TGS2610-C00, 3—TGS2611-C00, 4—TGS2612-D00, 5—TGS2611-E00, 6—TGS2620-C00, 7—TGS2602-B00, 8—TGS2610-D00, T—DS18B20, Rh—HIH-4000.

A block diagram of the e-nose system and sensor array is shown in Figure 1. Sensors are arranged in a radial array shape. Pressure in the sensor chamber that induces airflow is caused by a membrane micro-pump. The air flows through the input port located in a central part of a gas sensor chamber cover. A small orifice made in the rear wall of the gas chamber enables proper distribution of flux over the sensors. The device is controlled by the main AVR Atmega128 microcontroller. Control options and measurement results are presented on a graphical display, 64 × 128 pixels in size, coupled with the touch panel. The measurement results are stored in a memory card or in a computer via a USB interface.

The gas sensor array was previously calibrated with the known concentrations of single chemical substances in order to validate the system. The material presented in the discussed article contains sensor calibration data in the atmosphere of the air over the sewage level in the SBR reactor operating in stable laboratory conditions. At a further stage, the proposed system will be applied under real conditions for SBR reactors operating in a WWTP on a technical scale.

The gas sampling line contains a Perma Pure DM-110-24 desiccant-membrane dryer with a Nafion tube (length 61 cm, diameter 2.7 × 2.1 mm), filled with granulated silica gel. Sampling flow rate was set at 200 mL/min.

The measurement was conveyed continuously for 60 days with a 1 Hz reading frequency, which gave a total of 5.1 × 10^5^ multidimensional results. Steady state responses of the gas sensors were recorded from the samples. The sensors were flushed with clean air during the decantation phase, when the chambers of the reactor were prepared for fill with a new sewage load. During measurement, the average temperature of gases inside the sensing chamber was equal to 35 °C (±2 °C) and relative humidity was about 20% (±5%). The increased temperature inside the chamber was caused by heaters built into the MOS sensors, which minimized the risk of humidity condensation.

For comparison with the standard physical-chemical measurements of wastewater quality, 1 min average values of signals were taken into account, usually 5 min before the end of the sedimentation phase. The values of the signals during this time period were stable and showed insignificant variation.

### 2.3. Standard Parameters

For the liquid phase of purified sewage standard measurements for the following physical-chemical parameters were conducted: COD, N-NO_2_, N-NO_3_, N-NH_3_, pH, turbidity, TSS, and VOC. Measurements of the COD, N-NO_2_, N-NO_3_, N-NH_3_and TSS were performed using a HACH DR2800 spectrophotometer. Analysis was based on the methodology developed by HACH-Lange appearing with cuvette tests. For turbidity assessment the Eutech Instrument TN-100 turbidimeter was used. The pH value was measured with a HACH HQ40D digital pH meter. Measurement of contamination took place at the end of sedimentation phase. The impact of WWTP on the environment is frequently associated with emission of VOC causing odour nuisance, therefore during the measurements VOC levels were also analyzed. The VOC analysis was conducted using the portable gas chromatograph Photovac Voyager, operating in VOC mode on column V with a PID detector. The air was sampled using a long probe, and the sampling time was 30 s.

### 2.4. Data Analysis

Artificial neural networks (ANN) were used for the analysis of data from the gas sensors array. Gas sensor arrays coupled with ANN are the most closely related to the human sense of smell. However, this is a far-reaching simplification of the neurobiological system. In many cases, where the input-output relations are not very sophisticated, only one layer of hidden neurons is sufficient [47,48]. In other cases it is reasonable to use additional hidden layers. There is a general suggestion by Nielsen [49] that the number of hidden neurons should be less than 2*n* + 1, where n is the number of inputs. A network with a small number of hidden neurons does not allow the modelling of complex input-output reactions, but on the other hand networks with many neurons in the hidden layers have weak generalization abilities.

Onkal-Engin [38] tested multilayer perceptron (MLP) networks with numbers of hidden neurons from three to one hundred [39]. Finally, for the classification of wastewater, the optimum network 12-2 × 12-1 (12 inputs, two hidden layers of 12 neurons, one output neuron), and the regression network predicting BOD with the architecture 12-3 × 9-1, were selected. In another study of odour concentration prediction, where 16 sensors were used, an MLP network with two hidden layers consisting of 19 and 14 neurons, and a radial basis function (RBF) network with only one layer consisting of 35 neurons [18], were applied. Sohn [28], to predict the concentration of odour using 32 sensors, chose two hidden layers of 20 neurons. The mentioned articles above show that for the activation functions of hidden neurons, sigmoid, hyperbolic tangent, and—for the output neurons—linear functions are used. For network training the most popular is the back-propagation algorithm.

**Figure 2.**
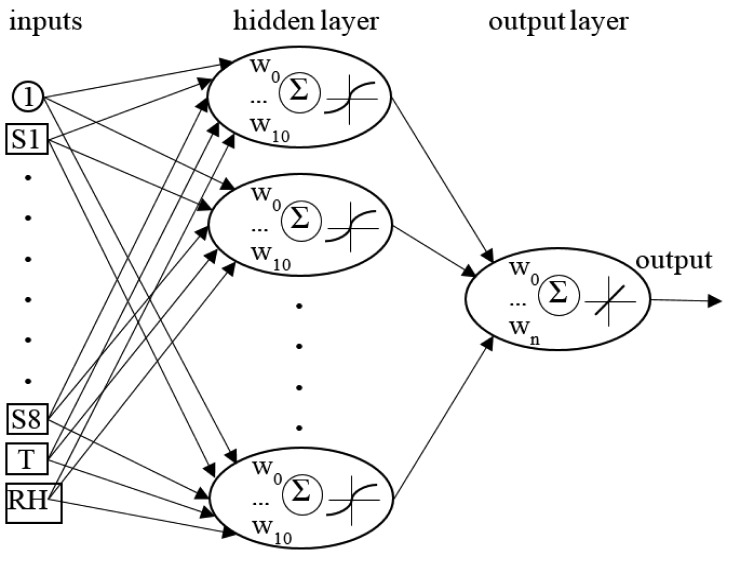
Architecture of neural network designed to predict wastewater quality.

For data analysis the feed forward artificial neural network MLP was used. The architecture of the net consisted of ten inputs (eight gas sensors and two sensors for temperature and relative humidity), one hidden layer with n-neurons, and one output neuron. The architecture (Figure 2) of the net was determined in order to maximize the generalization ability of a net at minimal complexity. The learning of a network is a process of iterative adjustment of weights in order to minimize net output error. Initial values of weights were set randomly with normal, standardized distribution: mean *w̄_ij_* = 0, variance *s* = 1. To learn the Broyden-Fletcher-Goldfarb-Shanno (BFGS) process, an iterative algorithm was applied. From the entire dataset, the learning (70%), testing (15%) and validation (15%) subsets were randomly chosen. The testing subset was used to examine network error during learning process. The validation subset was used for quality assessment of the learned net. The measuring system was not validated with the data obtained from the completely different experiment. The sum of squares (SOS) was used to evaluate the network output error (Equation (1)):

(1) $$E_{\text{SOS}}=\sum_{i=1}^{N}{\left(y_{1}-t_{i}\right)}^{2}$$

where *N* amount of data cases used to learning, *y_i_* prediction of network, *t_i_* expected value.

As an activation function of hidden and output neurons, a linear function was used: (identity) *x*, logistic sigmoid 1/(1 + *e*^−^*^x^*), hyperbolic tangent (*e^x^* − *e*^−^*^x^*)/(*e^x^* + *e*^−^*^x^*) and exponential *e*^−^*^x^* where *x*—sum of weighted input signals. Due to the fact that activation function has a limited range of a linear or nonlinear transformation range, the appropriate normalizing of input data should be applied. If not, the network output would be latched at the extreme value (saturation at the asymptotes). Normalization was performed by linear scaling of a minimum value of 0 and maximum value of 1 [50,51].

## 3. Results and Discussion

**Figure 3.**
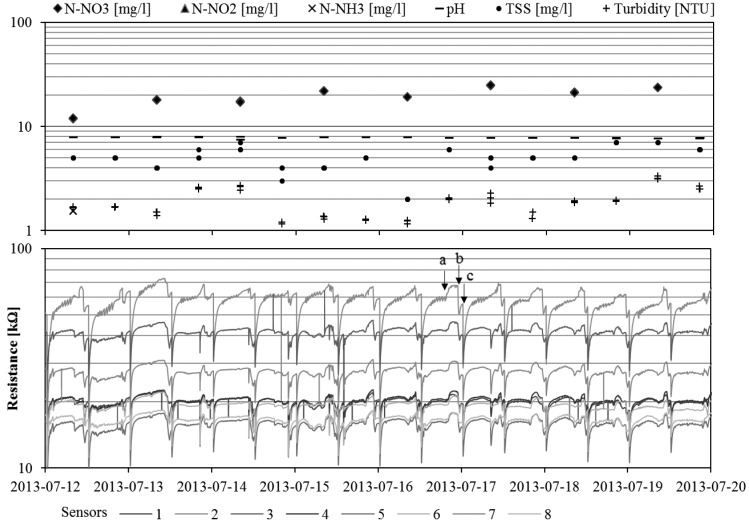
Results of one-week measurements from both the standard measurement (**upper chart**) and gas sensor array (**lower plot**), a—start of sedimentation, b—raw sewage load, c—beginning of aeration.

The summary of the standard physical-chemical parameters value obtained during measurements of normal SBR performance is presented in Table 4. All parameters are characteristic for treated municipal sewage. For many of the investigated parameters, the high scattering of data was not recorded, in particular for pH where the usable range is only around 1.15. Additionally, in Figure 3, a diagram that compares the readouts obtained using both applied techniques in the time domain (for a particular time period, to increase the readability of the figure) is presented.

Monitoring using the e-nose was conducted with a continuous mode and covered all phases of the wastewater treatment process cycle in SBR, except the decantation chase. During this particular phase, the sensor matrix was flushed with clean air from the laboratory. However, differences between the signal levels of the sedimentation phase and flushing period (convergent in time with a decantation phase) are not significant, which suggests that the air of the analyzed headspace was quite clean. The phases of bioreactor function were visualized, as in Figure 3. A narrow time range is selected to increase the readability of the recorded gas sensors array signals. For the discussed phases of experiment (sedimentation and flushing) the highest level of sensor resistance was achieved. At the moment of aeration switch-on an increased emission of pollutants into the gas phase occurs, which results in a rapid decrease in the resistance of the sensors in the matrix. Generally, there is a noticeable repetitive signal pattern for all gas sensors in all SBR working cycles.

The classic determinations of purified wastewater were discrete (point mode) at the end of each working period of the reactor, before termination of the sedimentation phase. Because the time interval between the end of the sedimentation phase and these sampling series was very short, the obtained results should be treated as the description of the last moments of the sedimentation and not a description of the decantation phase. As a result, it is not possible to precisely compare the readouts at a particular time-step. Due that to calculate the correlation between the measurements and e-nose readouts only the results of the sedimentation phase were used.

### 3.1. Determination of Hidden Neurons Number

**Figure 4.**
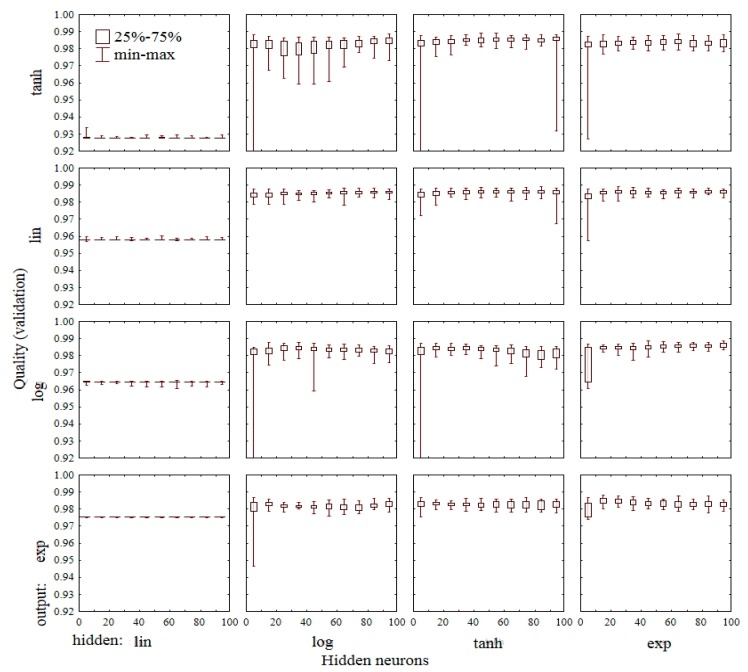
Quality value of network validation considering number of neurons in hidden layer *n* ∈ <1 ÷ 100> and different transfer function of hidden and output layer for COD prediction.

In order to determine the best parameters of artificial neural networks (number of hidden neurons, activation functions of hidden neurons, and output) to predict particular wastewater quality parameters, ten thousand networks with one hidden layer consisting of 1 to 100 hidden neurons were tested. To activate the hidden and output neurons, the linear (*lin*), logistic sigmoid (*log*), hyperbolic tangent (*tanh*), and the exponential (*exp*) functions were used. Figure 4 shows a boxplot of the COD prediction quality for each combination of activation f., and different numbers of hidden neurons. To assess the quality of COD prediction a validation data subset was taken, which was not used to learn the neural network. The bottom and the top of the box depict the first and the third quartiles. The whisker boundaries represent the minimum and maximum of the data of the assumed range. The highest average value of quality *Q̄_V_* = 0.986 has the network with *tanh* f. for activation of the hidden neurons and a *lin* f. for the output neuron. Other best networks have the activation functions of the hidden/output layer: *tanh*/*lin*, *exp*/*lin*, *exp*/*log* (all *Q̄_V_* = 0.985 ÷ 0.983). The worst networks have the activation functions *lin*/*lin*, *lin*/*log*, *lin*/*tanh* (*Q̄_V_* = 0.928 ÷ 0.965). It can be discerned that networks with neurons in the range 1 ÷ 10 have significantly lower values of validation quality. Networks with neurons in the range 10 ÷ 20 are almost identical to the networks with a higher number of hidden neurons. These results indicate that application of a large number of hidden neurons is not reasonable. Therefore for further inquiries one hidden layer with 20 neurons was adopted.

**Figure 5.**
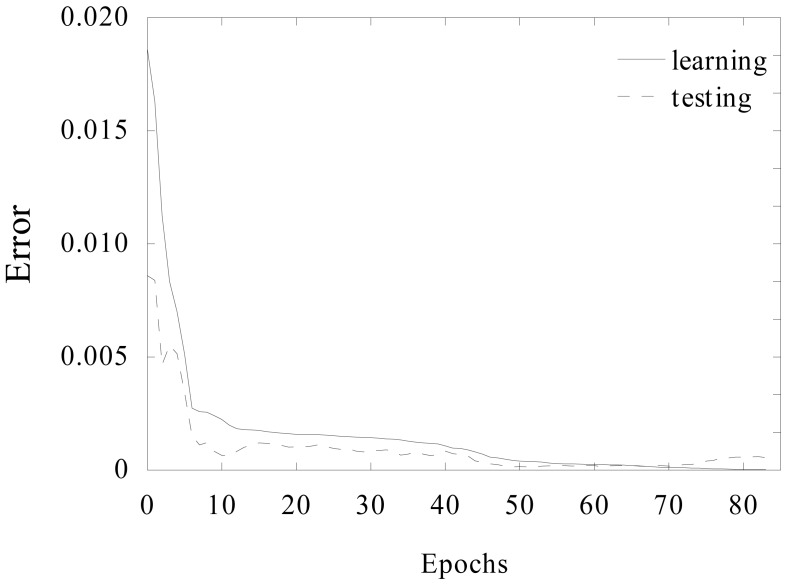
Example plot of net error alteration during learning process.

An example plot of network error during the learning process is shown in Figure 5. Network learning was terminated when output error for the testing subset was greater than the error for the learning subset. For most of the applied networks it was enough to use less than 100 learning epochs.

### 3.2. Determination of Activation Functions

Table 5 shows the mean values of neural network validation quality with 20 hidden neurons, considering different combinations of activation functions for each of wastewater quality parameters. The highest values for each parameter of treated wastewater quality are indicated in bold font.

Considering the hidden neurons, the highest scores frequently pertain to the following functions: *tanh* (five cases), subsequently *log* and *exp* (two cases each). The obtained results show that *lin* f. for hidden neurons cannot properly render nonlinear input-output relations. The neural network with the linear activation function of a hidden layer had predominantly the lowest values. Considering the output neurons, the highest scores were obtained by *lin* and *log* functions (three cases each), and then by *tanh* and *exp* functions (one case each).

To simplify the implementation of network parameters in the e-nose evaluation algorithm, the *tanh* f. for activation of the hidden layer and *lin* f. for activation of output neurons for each network were chosen. Additionally, for all networks a BFGS learning algorithm and SOS error function were applied.

### 3.3. Prediction of Wastewater Quality Parameters

**Figure 6.**
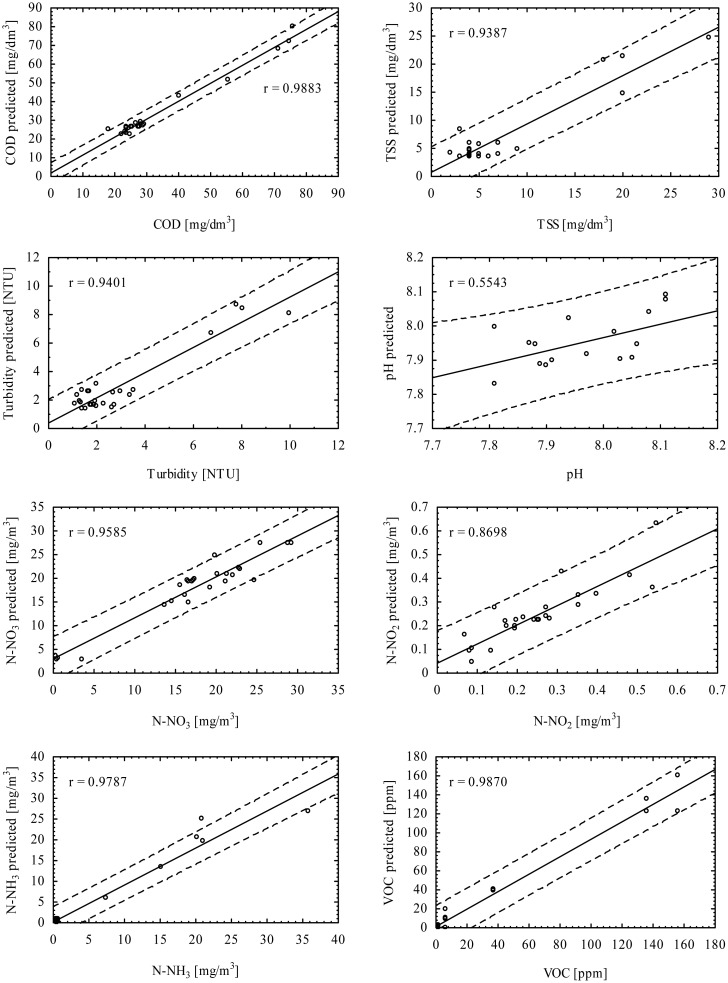
Correlation and confidence interval (0.95) between measured parameters and those predicted for validation subsets using e-nose: COD, TSS, turbidity, pH, N-NO_3_, N-NO_2_, N-NH_3_, VOC.

For prediction of particular purified wastewater quality parameters such as COD, TSS, turbidity, pH, N-NO_3_, N-NO_2_, N-NH_3_ and VOC the single MLP 10-1 × 20-1 (*tanh*/*lin*) networks for each parameter were chosen. The results of prediction are shown in the diagrams of Figure 6. Between the measured and predicted values of each parameter a regression line and 0.95 confidence interval was determined. The observed linearity of the measurements indicates that the values of parameters for the particular samples were below the saturation threshold of the sensors. Saturation of sensors occurs when all chemisorbed oxygen on the surface reacts with molecules of highly concentrated reducing gaseous compounds [17,52]. Additionally, the narrow range of the predicted parameters contributes to a high correlation coefficient. Insignificant correlation was observed for pH (*r* = 0.5543). For this parameter a wide confidence interval of around 0.3 is noticeable, which gives 60% of the measured pH scale. The best correlation (*r* = 0.9883) and narrow confidence interval about 10 mg/dm^3^ (17% of measured scale) was obtained for COD.

Turbidity and pH are indirectly related to other sewage parameters including the amount of biodegradable organic substances, often expressed as BOD or as COD of a suitable fraction. A high coefficient of correlation was achieved because the SBR reactor was working at stable conditions of temperature, oxygen concentration, and precise volume homogenization. Furthermore, the sewage with a stable composition, collected from a big settlement unit was dosed precisely, and schedule of service was rigorously obeyed.

For each neural network parameters including shift and scale were stored, to normalize input and output signals, input weights of particular neurons, and activation functions. Such parameters of learned networks as COD, TSS, turbidity, pH, N-NO_3_, N-NO_2_, N-NH_3_ and VOC can be implemented in a pattern recognition algorithm inside the e-nose. The recognition process does not require high computational power as network learning does, and can be realized by the standard 8-bit single-chip microcontroller.

The obtained dependences are only applicable for laboratory bioreactor and sources of wastewater under scrutiny. To implement the obtained characteristics in other processes, WWTP, or sewage types, more research into extrapolating the findings is required [17].

A prerequisite for comparing the results of the research is an identical protocol precisely defining the conditions, schedule and methods of measurement. As shown through research conducted by Fenner and Stuetz [53], the reproducibility of the results of tests with quiescent water samples is characterized by a large error of 10 to 15 of the percentage relative standard deviation (%RSD). There is a need to optimize the research protocol, especially relating to the times of the various phases of the measurement. The best match can be achieved with the precise timing of chamber and matrix sensor cleaning, sampling and time equilibration, and data acquisition. Using established protocols, the authors were able to achieve the order of 2 to 4%RSD [53]. In this paper there were described the measurements conducted using a continuous sampling headspace. The shape of signal readouts of sensor array and values of recorded resistance indicate (Figure 3), that the results prove satisfactory repeatability. The percentage relative standard deviation for the signals representing the treated wastewater (at the end of the sedimentation phase) was 8% to 19% RSD. The accuracy of the results is comparable with that of those obtained during an experiment using quiescent water sampling, as described in publication [53]. Percent relative standard deviation (%RSD) for the particular sensor signals recorded at the end of sedimentation phase from whole experiment is presented in Table 6.

## 4. Conclusions

It can be stated, on the basis of the research presented in this publication, that the proposed e-nose system, which consists of: (I) non-selective gas sensor array; (II) signal processing systems; and (III) trained artificial neural network (considered to be a multidimensional pattern recognition engine) can be successfully used to monitor the conformity of the effect of wastewater treatment by activated sludge in an SBR.

The following sensors were used to construct the e-nose system: TGS2600-B00 general air contaminants; TGS2602-B00 general air contaminants (high sensitivity to VOC and odorous gases); TGS2610-C00 butane and LP gas; TGS2610-D00 butane and LP gas (carbon filter); TGS2611-C00 methane and natural gas; TGS2611-E00 methane and natural gas (carbon filter); TGS2612-D00 methane, propane, isobutane and solvent vapours; TGS2620-C00 alcohol and solvent vapours; and a Dallas DS18B20 for temperature and Honeywell HIH-4000 for relative humidity measurements.

Each neural network for assessment of wastewater parameters is formed from ten signal inputs, a single hidden layer with 20 neurons, and one output for particular parameters. For activation of the hidden and output neurons the hyperbolic tangent and linear functions were used, respectively. The validation of the investigated system showed high correlation between the e-nose readouts and the parameters of the process occurring in stable treatment conditions in a laboratory batch reactor: COD (*r* = 0.988), TSS (*r* = 0.938), turbidity (*r* = 0.940), pH (*r* = 0.554), nitrogen compounds: N-NO_3_ (*r* = 0.958), N-NO_2_ (*r* = 0.869), N-NH_3_ (*r* = 0.978), and VOC (*r* = 0.987).

## Figures and Tables

**Table 1.** Review of environmental sources of air pollution malodour evaluated using e-nose.

**Source of Malodours**	**Type of Sensor; Sampling; Measurement**	**Analysis Method**	**Description**	**Reference**
landfill gas odors	16 tin oxide sensors; 20 L/min; meas. 90 s	ANN (35 neurons in hid. layer)	high correlation of particular sensors with odour concentration, low prediction network error (MSE) for MLP 0.000410 and RBF 0.000755 in the range of 0 to 200 ou_E_/m^3^	[18]
landfill site	3 × EOS835; 6 thin film MOS; 180 mL/min; 3 min meas./12 min recovery [19]	PCA	quantification of time percentage when the presence of odours is perceived at the landfill boundaries and near vicinity, comparison of e-nose data with meteorological measurements	[20]
waste disposal, landfill areas	6 to 8 tin oxide sensors	multilinear regression	correlation (*R*^2^ = 0.88) response of TGS822 sensor with odour concentration in the range of 0 to 1500 ou_E_/m^3^	[21]
waste incineration plant	6QMB; 400 mL/min absorption, 40 mL/min desorption;	PCA	correlation of particular sensor response with odour concentration in the range of 0 to 500 ou_E_/m^3^, detection of charcoal filter conditions	[22]
composting plant	6 MOS; 200 mL/min	PCA	Correlation of e-nose response with odour concentration in the range of 0 to 1500 ou_E_/m^3^, classification of air contamination from compost hall	[23]
composting plant	EOS835 25; 6MOS; 3 min meas./12 min recovery	PCA	96.4% classification accuracy of quantitative recognition test of odour source, high correlation (*R* = 0.95) e-nose response with odour concentration in the range of 20 to 50 ou_E_/m^3^	[24]
composting plant	EOS3; 6MOS; EOS9; 6MOS; 3 min meas./9 min recovery	PCA, Fourier	72% classification accuracy of quantitative recognition test of odour source, high correlation (*R* = 0.89) e-nose response with odour concentration in the range of 0 to 100 ou_E_/m^3^, response of e-nose compliant with human perception of nuisance	[25]
poultry farm	12 sensors (MOS, hybrid, tin dioxide, tungsten oxide)	ANN	accurate odour strength prediction with e-nose (*R* = 0.93)	[26]
poultry shed	24 MOS, 500 mL/min	PCA, PLS	high correlation of e-nose with odour concentration (*R* = 0.94) in the range of 0 to 4000 ou_E_/m^3^	[27]
piggery building	Aromascan A32S; 32CP	PCA, PLS, ANN (Matlab)	correlation of e-nose with odour concentration PCA (*R*^2^ = 0.44), PLS *R*^2^ = 0.79, ANN *R*^2^ = 0.62 with, estimation of biofilter efficiency	[28]
swine manure storage	Aromascan A32S; 32 CP	ANN 12 hid. neur. (NeuroShell2)	low correlation (*R*^2^ = 0.46) e-nose with odour concentration in the range of 0 to 5000 ou_E_/m^3^	[29]
piggery effluent ponds	Aromascan A32S; 32 CP	PCA, ANN (20 hid. neur., Matlab)	high correlation of e-nose with odour concentration (*R =* 0.98) in the range of 0 to 90 ou_E_/m^3^	[30]
cattle slurry	Odourmapper; 20 CP (polyindol); 100 mL/min; Aromascan A32S; 32 CP (polypyrrole); 100 mL/min	PCA (Genstat)	correlation of average e-nose response with odour concentration in the range of 0 to 1000 ou_E_/m^3^	[31]
livestock waste	20× CP polypyrrolee	PCA	discrimination of different odours	[32]
rendering plant	6QMB + 6MOS + O_2_ + H_2_O + CO + CO_2_; 3 s sample pulse, 60 s post-sampling measurement	PCA, PLS	sufficient correlation of e-nose with odour concentration in the range of 1000 to 30000 ou_E_/m^3^; biofilter controls	[33]
buildings material	KAMINA; 38 oxide gas sensor on chip	LDA, PLS	discrimination of different materials, correlation of e-nose response with perceived smell intensity in the range of 0 to 16 π (π is comparative unit determined on basis acetone vapours)	[34]

PCA—principal component analysis, CP—conducting polymer, MOS—metal oxide semiconductors, PLS—partial least squares, ANN—artificial neural network, QMB—quartz crystal microbalance, TON—threshold odour number, CCA—canonical correlation analysis, DFA—discriminant function analysis, LDA—linear discriminant analysis, ou_E_/m^3^—European odour unit per cubic meter.

**Table 2.** Application of e-nose for evaluation of wastewater parameters.

**Parameters**	**E-Nose; Sensors; Sampling; Measurement**	**Analysis Method**	**Description**	**Reference**
COD, TSS, VSS, turbidity	FOX3000 (Alpha M.O.S.), 12 MOS; sampling 150 mL/min; injection time 60 s	PCA	Poor correlation between e-nose response and VSS (*R* = 0.67), weak correlation with other parameters: CODtot (*R* = 0.53) CODsol (*R* = 0.36) TSS (*R* = 0.52), turbidity (*R* = 0.53)	[17]
detection of pollutants	eNOSE 5000, ProSat, Marconi Applied Technologies, conducting polymer sensors, 40 s purge, 1 min. sampling, 3 min 20 s de-purge	PCA	Real-time detection of unknown pollutants, intermittent or accidental discharge	[35–37]
BOD, classification of wastewater respecting odour	Neotronics Scientific Ltd. model D; 12 CP (polypyrrole); 600 mL/min, odour profiles at 1 min	Multivariate discriminant, canonical correlation, ANN	classification of wastewater, correlation of e-nose response with BOD	[38]
BOD, classification of wastewater respecting odour	Neotronics Scientific Ltd. model D; 12 CP (polypyrrole)	canonical discriminant and correlation	classification of wastewater, correlation of e-nose response with BOD	[39]
wastewater type discrimination	Pen-2 (WMA Airsense Analysentechnik, 10 MOS) 400 mL/min; 50 s reference air, 50 s sampling Cyranose 320 (Cyrano Sciences, 32 CP) 50 s reference air, 50 s sampling;	PCA	Discrimination between odour samples from different locations in WWTP	[40]
odour concentration	Neotronics Scientific Ltd. model D; 12CP (polypyrrole); 600 mL/min, odour profiles at 1 min	CCA	correlation between TON and e-nose in the range of 125 to 781,066 ou_E_/m^3^	[41]
odour concentration	5× e-nose; 6 MOS	supervised modelling, DFA	assessment of odour annoyance in vicinity of waste treatment plant using e-nose compared with meteorological measurements, correlation e-nose with odour concentration in the range of 0 to 4000 ou_E_/m^3^	[42]
odour concentration	Airsense PEN2; 10 MOS	ANN, DFA, PCA	correlation e-nose with odour concentration ranges from 0 to 200 ou_E_/m^3^ (*R*^2^ = 0.94), discrimination between different samples from biofilters	[43]
classification of wastewater respecting odour, odour concentration	EOS25, EOS28, EOS35; 3 min meas./12 recovery	PCA	classification of odour sources with high accuracy (*R* = 0.95 for samples 100 to 150 ou_E_/m^3^), high correlation (*R* > 0.9) e-nose response with odour concentration in the range of 20 to 80 ou_E_/m^3^	[44]

**Table 3.** Overview of the gas sensors implemented in the e-nose [46].

**Type**	**Description**	**Detection Range [ppm]**	**Heating Element:**	**Sensing Element:**	**Sensitivity (Change Ratio)**

			**Voltage Resistance Current Power**	**Voltage Load Resistance Power Resistance in Gas**	
TGS2600-B00 Figaro	general air contaminants	1–30 (H_2_)	5.0 ± 0.2 V	5.0 ± 0.2 V	0.3–0.6 for $\frac{Rs(10\text{ppm}H_{2})}{Rs(\text{air})}$
			83 Ω	>0.45 kΩ	
			42 ± 4 mA	<15 mW	
			210 mW	10–90 kΩ clean air	

TGS2602-B00 Figaro	general air contaminants (high sensitivity to VOC and odorous gases)	1–30 (EtOH)	5.0 ± 0.2 V	5.0 ± 0.2 V	0.15–0.5 for $\frac{Rs(10\text{ppm EtO})}{Rs(\text{air})}$
			59 Ω	>0.45 kΩ	
			56 ± 5 mA	<15 mW	
			280 mW	10–100 kΩ clean air	

TGS2610-C00 Figaro	butane. LP gas	500–10,000	5.0 ± 0.2 V	5.0 ± 0.2 V	0.56–0.06 for $\frac{Rs(3000\text{ppm})}{Rs(1000\text{ppm})}$
			59 Ω	>0.45 kΩ	
			56 ± 5 mA	<15 mW	
			280 mW	0.68–6.8 kΩ iso-butane	
				1800 ppm	

TGS2610-D00 Figaro	butane. LP gas (carbon filter)	500–10,000	5.0 ± 0.2 V	5.0 ± 0.2 V	0.56–0.06 for $\frac{Rs(3000\text{ppm})}{Rs(1000\text{ppm})}$
			59 Ω	>0.45 kΩ	
			56 ± 5 mA	<15 mW	
			280 mW	0.68–6.8 kΩ iso-butane	
				1800 ppm	

TGS2611-C00 Figaro	methane. natural gas	500–10,000	5.0 ± 0.2 V	5.0 ± 0.2 V	0.6–0.06 for $\frac{Rs(9000\text{ppm})}{Rs(3000\text{ppm})}$
			59 Ω	>0.45 kΩ	
			56 ± 5 mA	<15 mW	
			280 ± 25 mW	0.68–6.8 kΩ methane	
				5000 ppm	

TGS2611-E00 Figaro	methane. natural gas (carbon filter)	500–10,000	5.0 ± 0.2 V	5.0 ± 0.2 V	0.6–0.06 for $\frac{Rs(9000\text{ppm})}{Rs(3000\text{ppm})}$
			59 Ω	>0.45 kΩ	
			56 ± 5 mA	<15 mW	
			280 ± 25 mW	0.68–6.8 kΩ methane	
				5000 ppm	

TGS2612-D00 Figaro	methane. propane. iso-butane, solvent vapors	1%–25% LEL	5.0 ± 0.2 V	5.0 ± 0.2 V	0.5–0.65 for $\frac{Rs(9000\text{ppm})}{Rs(3000\text{ppm})}$
			59 Ω	>0.45 kΩ	
			56 ± 5 mA	<15 mW	
			280 mW	0.68–6.8 kΩ methane	
				5000 ppm	

TGS2620-C00 Figaro	alcohol. solvent vapors	50–5,000	5.0 ± 0.2 V	5.0 ± 0.2 V	0.3–0.5 for $\frac{Rs(300\text{ppm})}{Rs(50\text{ppm})}$
			83 Ω	>0.45 kΩ	
			42 ± 4 mA	<15 mW	
			210 mW	1–5 kΩ ethanol 300 ppm	

**Table 4.** The range and average values of wastewater quality standard parameters measured during normal SBR performance, for which e-nose measurements were also conducted.

**Statistics**	**COD [mg/dm^3^]**	**TSS [mg/dm^3^]**	**Turbidity [NTU]**	**pH**	**N-NO_3_ [mg/dm^3^]**	**N-NO_2_ [mg/dm^3^]**	**N-NH_3_ [mg/dm^3^]**
min	17.1	2.0	1.0	7.08	0.3	0.07	0.2
max	75.8	30.0	10.2	8.23	29.3	0.90	35.8
mean	32.7	6.4	2.8	7.96	17.8	0.34	5.0
median	27.4	4.0	2.0	7.97	19.1	0.32	0.8

**Table 5.** Mean values of neural network validation quality with 20 hidden neurons, considering different combinations of activation functions for each of wastewater quality parameters. The highest values for each parameter of treated wastewater quality are indicated in bold font.

**Parameter**	**Activation of Output Layer**	**Activation of Hidden Layer**			

		***Lin***	***Log***	***Tanh***	***Exp***
COD	lin	0.958	0.984	0.985	0.986
	log	0.958	0.983	0.985	0.984
	tanh	0.928	0.978	**0.986**	0.983
	exp	0.975	0.982	0.982	0.985

TSS	lin	0.778	0.927	0.931	0.936
	log	0.894	0.888	**0.957**	0.893
	tanh	0.852	0.922	0.941	0.887
	exp	0.772	0.908	0.848	0.897

Turbidity	lin	0.814	**0.951**	0.939	0.942
	log	0.925	0.932	0.946	0.909
	tanh	0.869	0.924	0.914	0.921
	exp	0.826	0.947	0.923	0.942

pH	lin	0.441	0.452	0.517	0.510
	log	0.436	0.430	**0.627**	0.586
	tanh	0.439	0.544	0.581	0.605
	exp	0.463	0.471	0.571	0.444

N-NO_3_	lin	0.901	0.959	0.958	0.946
	log	0.910	0.949	0.959	0.927
	tanh	0.901	0.949	0.954	0.943
	exp	0.863	0.951	**0.961**	0.954

N-NO_2_	lin	0.609	0.858	0.861	**0.881**

	log	0.568	0.622	0.856	0.851

	tanh	0.618	0.847	0.866	0.770

	exp	0.633	0.484	0.868	0.869

N-NH_3_	lin	0.879	0.961	**0.974**	0.952
	log	0.879	0.938	0.923	0.969
	tanh	0.880	0.939	0.968	0.877
	exp	0.879	0.969	0.959	0.755

VOC	lin	0.989	0.987	0.987	0.984
	log	0.785	0.993	0.995	**0.998**
	tanh	0.995	**0.998**	0.997	0.997
	exp	0.977	0.974	0.991	0.978

**Table 6.** Percent relative standard deviation (%RSD) of response of particular sensors.

	**2600-B00**	**2602-B00**	**2610-C00**	**2610-D00**	**2611-C00**	**2611-E00**	**2612-D00**	**2620-C00**
Mean	20.86	53.25	42.51	20.30	16.51	20.24	27.70	17.37
Standard deviation	1.94	10.36	4.913	2.366	1.897	1.66	3.35	1.679
%RSD	9%	19%	12%	12%	11%	8%	12%	10%
